# High incidence of prostate cancer metastasis in Afro-Brazilian men with low educational levels: a retrospective observational study

**DOI:** 10.1186/1471-2458-13-537

**Published:** 2013-06-04

**Authors:** Alexandre Barbosa Câmara de Souza, Hugo Gonçalo Guedes, Victor Carbone Bernardes Oliveira, Fábio Aires de Araújo, Carlos Cesar Oliveira Ramos, Karina Carla Paula Medeiros, Raimundo Fernandes Araújo

**Affiliations:** 1 Department of Morphology, Federal University of Rio Grande do Norte, Natal 59072-970, Rio Grande do Norte, Brazil; 2 Doctor Luiz Antonio Hospital, Natal 59040-000, Rio Grande do Norte, Brazil

**Keywords:** Epidemiology, Prognosis, Prostate cancer, Risk factor, Educational levels

## Abstract

**Background:**

This study investigated factors related to ethnicity and educational level, their correlation with tumor stage at the time of diagnosis, and their influence on treatment outcomes in patients with prostate cancer.

**Methods:**

In this retrospective observational study, we analyzed the medical records of 1,349 male patients treated for prostatic adenocarcinoma. We collected information about sociodemographic variables, including educational level and self-reported skin color. We also classified the disease according whether it was to more likely to present with metastasis and measured the tumor response to treatment.

**Results:**

Less-educated (<8 years of education) individuals were 4.8 times more likely to develop metastasis than those with more education (>11 years of education; *p* < 0.001). Similarly, patients with a self-reported black skin color had a 300% increased risk of metastasis at diagnosis (*p* = 0.001). Distant metastasis was independently correlated with worse outcomes, such that individuals with distant metastasis were 10 times more likely to die than were those without distant metastasis.

**Conclusions:**

Patients with self-reported black skin color and <8 years of education were more likely to display advanced disease at the time of diagnosis compared with their counterparts. Only the presence of metastasis was independently associated with mortality or progressive disease.

## Background

Prostate adenocarcinoma is the sixth most prevalent cancer in the world and the most common cancer among men in Europe, North America, and parts of Africa. After non-melanoma skin cancer, prostate adenocarcinoma is the second most often diagnosed cancer and the third leading cause of cancer-related deaths in the United States (US) [1]. In addition, prostate adenocarcinoma is the second most prevalent cancer in Brazil, with an estimated 60,180 new cases in 2012. According to the National Cancer Institute of Brazil, the increase in incidence rates may be explained by increased life expectancy, improvement in and development of diagnostic methods, and improved quality of information systems in Brazil [2].

There are differences in prostate adenocarcinoma rates across ethnic groups. In the US, African-Americans have the highest disease rate compared with other ethnicities. Caucasians have an approximately 60% lower rate of prostate adenocarcinoma diagnosis than do African-Americans, and the prevalence is even lower among those with a Latin American or Asian origin [3]. Differential genetic susceptibility and socioeconomic and environmental factors likely contribute to the observed differences in incidence rates among ethnicities [4-7]. For example, obesity is one of the most studied factors in cancer. Obesity has been associated with increased exposure to carcinogens in the diet [8], increased cardiovascular risk [9], higher prevalence of high-grade prostate cancer (15.5%), and increased patient morbidity and mortality [10-13]. Thus, several factors are involved in prostate adenocarcinoma epidemiology, and the identification of useful prognostic factors for this neoplasm is extremely important for understanding the disease.

The most important clinical factors currently available are the prostate-specific antigen (PSA) level and histological differentiation degree in tumor biopsy fragments; these two markers are expressed as the Gleason score [14,15]. Although the Gleason score is valuable, its sole use does not define patient prognosis for prostate cancer [16]. Consequently, additional comprehensive studies of prognostic factors for prostatic adenocarcinoma are necessary to find the population that is most in need of attention and early diagnosis.

This study aimed to identify ethnic and educational levels as additional factors related to prostate adenocarcinoma, determine their correlation with tumor stage at the time of diagnosis, and elucidate their influence on patient outcomes.

## Methods

The institutional committees (No. 030/0030/2006) of the Liga Norteriograndense Contra o Cancer, Brazil, approved this research.

This transversal study included the retrospective analysis of mortality or progressive disease state in 1,349 male patients with biopsy-confirmed prostate adenocarcinoma (International Classification of Diseases, 10th edition, code C61). Patients were treated between 1997 and 2005 at Luis Antonio Hospital, a Brazilian Health System cancer referral center in Natal, Brazil. Natal is the capital of Rio Grande do Norte State in the northeastern part of the country and has a population of >1 million inhabitants. Trained researchers collected data, which included hospital and medical records, between August 2007 and May 2008.

From all cases, researchers gathered medical record data related to the variables of interest: patient age at cancer diagnosis, race/ethnicity, residential address at diagnosis, tumor stage and grade, deaths, age, and educational level (<8 years = incomplete elementary school; >11 years = high school completion). Race/ethnicity was classified according to the mutually exclusive self-report for skin color (black: yes or no). After diagnosis, we defined the disease as either aggressive or nonaggressive. Regional or distant tumors based on imaging study results were classified as aggressive, while tumors confined to the prostate (localized) were nonaggressive. The endpoint was mortality or progressive disease at the end of the first stage of treatment (first consultation after hospital treatment, with 2 weeks to 1 month).

A chi-squared test determined the distribution differences of educational status and skin color. Multivariate logistic regression models examined the influence of socioeconomic factors on the presence of metastasis at diagnosis and patient outcome after the first stage of treatment. Regression analyses were conducted in two steps: Model 1 assessed the correlation of each socioeconomic variable with the presence or absence of metastasis at diagnosis, and Model 2 adjusted education status and skin color for the presence of metastasis to determine the impact of these variables on mortality. The analyses showed no colinearity. All statistics were performed using SPSS software (ver. 13.0 for Windows; SPSS Inc., Chicago, IL, USA) with a significance level of *p* < 0.05.

## Results

Table 1 shows the descriptive data of the study participants.

**Table 1:** Descriptive data

**Variable**	***n***	**%**	**Mean**	**SD**
**Age (years)**			**71.25**	**9.10**
≤50	**23**	**1.7**		
51 − 60	**151**	**11.2**		
61 − 70	**401**	**29.7**		
71 − 80	**562**	**41.7**		
81 − 90	**205**	**15.2**		
>90	**7**	**0.5**		
**Educational level (years)**^Ξ^			**-**	**-**
<8	**504**	**66.9**		
>11	**249**	**33.1**		
**Self-reported skin color**^Ξ^			**-**	**-**
Black	**122**	**11.9**		
Other	**905**	**88.1**		
**Treatment**^Ξ^			**-**	**-**
Surgery	**190**	**14.9**		
Radiotherapy	**294**	**23.0**		
Chemotherapy	**26**	**2.0**		
Hormone therapy	**512**	**40.1**		
Multiple treatments	**254**	**19.9**		
**Metastasis**^Ξ^			**-**	**-**
Yes	**272**	**24.3**		
No	**849**	**75.7**		
**Mortality or progressive disease **^Ξ^			**-**	**-**
Yes	**608**	**77.9**		
No	**172**	**22.1**		

*SD* standard deviation.

^Ξ^categorical variables.

Univariate analysis showed a significant correlation between the presence of aggressive metastasis and educational levels (*p* < 0.001) and self-reported skin color (*p* = 0.006), suggesting that these variables are risk factors for distant metastasis at the time of diagnosis. Multivariate regression analysis confirmed these results and demonstrated independent correlations: the chance of developing an aggressive metastasis was increased 4.8-fold in patients with <8 years of education compared with those who had received >11 years of education (*p* < 0.001). Moreover, patients with a self-reported black skin color showed a 300% increase in aggressive metastasis compared with patients of other skin colors (*p* = 0.001).

Although self-reported skin color was not associated with mortality or progressive disease, low educational levels (*p* = 0.002) and the presence of metastasis (*p* < 0.001) were positively associated with the endpoint. The results from the multivariate regression analyses demonstrated that only the presence of aggressive metastasis was independently correlated with a negative outcome, such that patients with distant metastasis were 10 times more likely to die (Table 2).

**Table 2:** Multivariate regression analyses

			**Metastasis**			**Mortality or progressive disease**
	**Yes (%)**	***p***	**OR (95% CI)**	**Yes (%)**	***p***	**OR (95% CI)**
**Educational level (years)**		<0.001	4.778 (2.821–8.093)		0.148	
<8	104 (25.2)			194 (6.7)		
>11	24 (10.4)			124 (89.2)		
**Self-reported skin color**		0.001	3.053 (1.552–6.004)		**-**	**-**
Black	15 (13.4)			72 (84.7)		**-**
Other	190 (25.2)			428 (79.3)		
**Metastasis**		**-**	**-**		<0.001	10.026 (6.187–16.247)
Yes	272 (100.0)		**-**	80 (44.0)		
No	0 (0.0)			489 (90.6)		

*OR* odds ratio, *CI* confidence interval.

## Discussion

The inaccurate prediction of treatment outcomes for patients with prostate cancer is a public health problem because different treatment options are associated with different morbidity rates. For example, erectile dysfunction affects 80% of patients who have undergone radical prostatectomy [16]. The identification of additional prognostic factors will thus help physicians select the most appropriate treatment for each case.

Our results that a lower educational level was related to an increased likelihood of having a metastasis at the time of diagnosis may be due to decreased healthcare use among less-educated patients. Individuals who do not seek treatment or do not have access to healthcare may receive a delayed diagnosis and be less likely to adhere to treatment regimens. In contrast, well-educated individuals are more likely to undergo timely screening (PSA testing) and urological monitoring. A study conducted in Sao Paulo State in Brazil found that 44% of 992 men with a low socioeconomic status had never received a preventive examination for prostate adenocarcinoma; this pattern was most prevalent among men aged <70 years, those with a black or brown skin color, and those with <9 years of education [17]. Another study in Sao Paulo showed high mortality rates among men living in poor areas, which may be related to a lack of access to preventive cancer screening [18]. In addition, a significant association between low educational level and prostate cancer incidence was demonstrated in a cohort from a US study. Although these subjects were followed for an average of 8.2 years, a strong association with cancer dissemination was not found [19].

Individuals with African descent have been associated with higher prostate cancer incidence rates and worse prognoses compared with individuals of Caucasian ethnicity. A United Kingdom study found that the incidence of prostate cancer was 166/100,000 among black men (black Caribbean, black Africans, and black unclassified), but only 56.4/100,000 among white men [20]. This difference in incidence rates has also been documented in the US, where African-American men show a higher incidence of prostate cancer, have a 2-fold greater likelihood of future disease development, and display twice the mortality risk compared with Caucasian men [21,22].

Based upon previous findings and our current results, African descent and a low educational level may have interacting effects on prostate cancer treatment outcomes. In several countries throughout the world, individuals of African descent receive less education than do other ethnic groups, such as Caucasians; however, studies have shown that factors related to the lack of education alone cannot explain the treatment outcomes associated with this group. It is possible that genetic factors inherent to this population, including the expression of genes related to androgen activity, affect the presentation of prostate cancer. For example, young men of African descent have higher testosterone levels and exhibit changes in androgen receptors. These factors may account for the higher incidence of prostate cancer and worse treatment outcomes observed in this group, excluding the impact of socioeconomic effects [23,24]. Therefore, while educational level and genetic predispositions influence the development and treatment of prostate cancer in individuals of African ethnicity, the interaction between these variables may have an even greater impact on disease outcomes.

We acknowledge that this study has limitations. First, this was a retrospective study, and potential confounding effects of complex variable relationships (i.e., PSA screening, Gleason score, and number of prior physician visits) may have limited our power of analysis. Unfortunately, these variables could not be analyzed. Second, there may have been variations or inaccuracies in patient self-reported information, which may have biased our results. However, we believe that the large sample size likely negated, or at least minimized, possible bias.

## Conclusions

This study demonstrated that men of African descent and those with <8 years of education had a greater chance of displaying advanced disease at the time of diagnosis compared with patients of other skin colors and higher educational level (>11 years). These findings should guide improvements in the public health system to change these patterns. Our results show that this population deserves special attention to ensure early diagnosis and treatment as well as improved health outcomes. Further research should be conducted to better understand these variables, other related factors, and the relationships among genetic and sociodemographic influences to improve prognostic classification in the Brazilian population.

## Competing interests

The authors declare that they have no competing interests.

## Authors’ contributions

ABCS and HGG participated in the study design, performed statistical analyses, and drafted the manuscript. VCBO and FAA participated in the study design and drafted the manuscript. CCOR conducted the prostate cancer biopsies. KCPM helped design the study. RFAJ conceived the study, participated in the study design, and coordinated and drafted the manuscript. All authors read and approved the final manuscript.

## Authors’ information

ABCS, HGG, VCBO and FAA are Medicine Doctors graduates of the Federal University of Rio Grande do Norte; CCOR is Pathologist of the Doctor Luiz Antonio Hospital; KCPM is Professor of Biomedicine of the Federal University of Rio Grande do Norte and RFAJ is PhD, Professor of Medicine of the, Federal University of Rio Grande do Norte.

## Pre-publication history

The pre-publication history for this paper can be accessed here:

http://www.biomedcentral.com/1471-2458/13/537/prepub
